# Leukocyte Dynamics Reveal a Persistent Myeloid Dominance in Giant Cell Arteritis and Polymyalgia Rheumatica

**DOI:** 10.3389/fimmu.2019.01981

**Published:** 2019-08-22

**Authors:** Yannick van Sleen, Jacoba C. Graver, Wayel H. Abdulahad, Kornelis S. M. van der Geest, Annemieke M. H. Boots, Maria Sandovici, Elisabeth Brouwer

**Affiliations:** Vasculitis Expertise Centre Groningen, Department of Rheumatology and Clinical Immunology, University of Groningen, University Medical Centre Groningen, Groningen, Netherlands

**Keywords:** giant cell arteritis, polymyalgia rheumatica, vasculitis, glucocorticoids, longitudinal cohort study, relapses, treatment-free remission

## Abstract

Giant cell arteritis (GCA) and polymyalgia rheumatica (PMR) are inflammatory diseases requiring long-term glucocorticoid treatment. Limited data on dynamics in leukocyte counts before, during and after treatment are available. Leukocyte counts were measured, as cellular markers of inflammation, at fixed time points in our prospectively studied cohort of pre-treatment glucocorticoid-naive GCA (*N* = 42) and PMR (*N* = 31) patients. Values were compared with age-matched healthy controls (HCs; *N* = 51) and infection controls (*N* = 16). We report that before start of treatment monocyte and neutrophil counts were higher in GCA and PMR patients than in HCs, while NK- and B-cell counts were lower. C-reactive protein (CRP) levels correlated positively with monocyte counts in GCA, and negatively with B-cell and NK-cell counts in PMR. During glucocorticoid treatment, myeloid subsets remained elevated whereas lymphoid subsets tended to fluctuate. Interestingly, erythrocyte sedimentation rate (ESR) outperformed CRP as marker for relapses in GCA. We defined stable treatment-free remission groups in both GCA and PMR. GCA patients in treatment-free remission still demonstrated elevated monocytes, neutrophils, ESR, and platelets. PMR patients in treatment-free remission had normalized levels of inflammation markers, but did have elevated monocytes, lowered CD8+ T-cell counts and lowered NK-cell counts. Finally, we showed that low hemoglobin level was predictive for long-term GC treatment in PMR. Overall, leukocyte composition shifts toward the myeloid lineage in GCA and PMR. This myeloid profile, likely induced by effects of inflammation on hematopoietic stem cell differentiation, persisted during glucocorticoid treatment. Surprisingly, the myeloid profile was retained in treatment-free remission, which may reflect ongoing subclinical inflammation.

## Introduction

Giant cell arteritis (GCA) and polymyalgia rheumatica (PMR) are aging-related inflammatory diseases that frequently overlap (1). GCA and Takayasu arteritis both belong to the large vessel vasculitis, but GCA only occurs in the elderly and can also affect cranial arteries. Involvement of cranial arteries (C-GCA) is associated with cranial symptoms including headache, jaw claudication, and vision loss. Large vessel GCA (LV-GCA) is more difficult to diagnose due to non-specific symptoms such as weight loss and low-grade fever. PMR is characterized by bursitis and synovitis leading to pain and stiffness mainly in the shoulder and hip girdle (2). PMR is diagnosed in up to 60% of GCA patients (3), implying overlapping pathogenic pathways. The pathogenesis of GCA, and especially PMR, are incompletely understood (4). In GCA, temporal artery biopsies (TABs) reveal a granulomatous infiltrate of macrophages and CD4+ T-cells in the vessel wall (5, 6). Infiltrating B-cells and neutrophils have been found in lower numbers (7–10). The vast majority of newly diagnosed GCA and PMR patients display elevated interleukin (IL)-6-dependent acute-phase markers such as ESR and CRP (11, 12). From the 1950s until now, glucocorticoids (GCs) have remained the cornerstone of treatment in GCA and PMR (13). GC treatment, however, is accompanied by side-effects, and relapses during GC treatment are common (14, 15). More recently, progress has been made regarding GC-sparing therapies in GCA and PMR (16–18). The effects of GC-mediated immunosuppression are pleiotropic and not yet completely understood (19). GCs strongly repress the acute-phase response (17), and therewith repress the utility of CRP, ESR, and other inflammatory markers in monitoring patients during treatment. Furthermore, recent evidence suggests that GCA patients on GC treatment with a normal CRP/ESR and absence of symptoms can still have persistent vessel wall inflammation (20–22). Consequently, it is unknown whether patients who reached treatment-free remission are truly in remission or are suffering from ongoing subclinical disease. This is important, as GCA patients with subclinical vasculitis are at risk of aneurysm development and aortic dissection (1, 23). In search for cellular markers of inflammation in GCA and PMR, we documented leukocyte dynamics during the entire disease course. Previously, altered monocyte, neutrophil, and B-cell blood counts have been reported at diagnosis (9, 24, 25). In addition, small and mostly short-term studies have addressed the effect of GCs on blood leukocyte subset counts in GCA and PMR patients. Both myeloid [monocytes (25, 26), neutrophils (10, 24)] and lymphoid [CD4+/CD8+ T-cells (26–28), B-cells (9), NK-cells (26, 29)] cell counts appear to be affected by GCs. However, to the best of our knowledge, a comprehensive long-term study comparing leukocyte subset counts before, during and after GC treatment in GCA and PMR patients has not been performed. The current study was conducted in our prospective cohort in which glucocorticoid-naive GCA and PMR patients were requested to participate at diagnosis and were followed for up to 7 years. At fixed time points, leukocyte counts and other inflammatory markers were determined. We investigated the effects of disease on leukocyte subsets by comparison to healthy and infection controls. Next, we analyzed the effects of short- and long-term treatment on leukocyte subsets in GCA and PMR patients and extended our investigation to patients who had reached stable treatment-free remission. In addition, we evaluated the usefulness of leukocyte subsets and inflammatory markers in identifying relapses and assessed their prognostic value before start of treatment.

## Materials and Methods

### Patient Population

Characteristics of newly-diagnosed patients before start of treatment and characteristics of controls are displayed in Table 1. Forty-two GCA and 31 PMR patients participated in our cohort study and were seen at the Rheumatology and Clinical Immunology outpatient clinic of the University Medical Center Groningen between 2010 and 2018. These patients did not use GCs or other disease modifying anti-rheumatic drugs (DMARDs) at pre-treatment assessment. GCA patients were diagnosed based on a positive temporal artery biopsy (TAB) and/or a positive 18F-fluorodeoxyglucose-positron emission tomography-computed tomography (FDG-PET-CT) for LV-GCA. In GCA 29 of the 42 patients fulfilled the 1990 ACR criteria, as these criteria are mainly useful in diagnosis of C-GCA rather than LV-GCA. Diagnosis of PMR patients was based on a positive FDG-PET-CT scan, or based on clinical signs and symptoms if no FDG-PET-CT could be performed. Twenty-five out of 31 PMR patients fulfilled the Chuang criteria. Three PMR patients without a FDG-PET-CT scan, did not fulfill the Chuang criteria due to ESR levels below 40 mm/h, but did have elevated CRP levels (>10 mg/L). All but one PMR patient fulfilled the preliminary ACR/EULAR 2012 classification criteria (30). This patient did fulfill the Chuang criteria and had a positive FDG-PET-CT for PMR. This study included cross-sectional data of 51 age- and sex-matched HCs and also 16 age-matched INFs. HCs were screened for past and present morbidities. Hospitalized INFs who suffered from urinary tract infection (*n* = 10) or pneumonia (*n* = 6) were requested to participate. All INFs were recruited during active infection, up to 7 days after admission to the hospital. Volunteers in both control groups did not take any immunosuppressive drugs nor had comorbid diseases. Written informed consent was obtained from all study participants. All procedures were in compliance with the declaration of Helsinki. The study was approved by the institutional review board of the University Medical Center Groningen (METc2012/375 for HC and METc2010/222 for GCA, PMR, and INF).

**Table 1:** Pre-treatment characteristics of newly diagnosed, treatment-naive giant cell arteritis and polymyalgia rheumatica patients, aged healthy controls, and aged infection controls.

	**HC**	**GCA**	**PMR**	**INF**	***p*-value HC vs. GCA**	***p*-value HC vs. PMR**	***p*-value HC vs. INF**
*n*	51	42	31	16	–	–	–
Age in years; median (range)	**72** (57-91)	**72** (52-89)	**73** (54-84)	**74** (47-97)	NS	NS	NS
Females (%)	32 (63)	28 (67)	20 (65)	5 (31)	NS	NS	0.043
Smoking status; smoking/non-smoking	9/42	13/29	3/28	4/10	NS	NS	NS
TAB positive/performed	NA	23/29	0/6	NA	–	–	–
FDG-PET-CT positive for GCA/PMR/GCA+PMR	NA	19/0/10	0/23/0	NA	–	–	–
IL-6 pg/mL; median (range)^*^	**1.5** (0.9–4.2)	**11.5** (1.4–233.6)	**19.8** (2-117)	**22.1** (0.9–152.7)	<0.0001	<0.0001	<0.0001
CRP mg/L; median (range)	**5** (0–7)	**47** (2.2–215)	**42** (3.2–186)	**70** (10-339)	<0.0001	<0.0001	<0.0001
ESR mm/h; median (range)	**10** (1-28)	**81** (7-121)	**57** (8-109)	**60** (10-118)	<0.0001	<0.0001	<0.0001
Hb mmol/L; median (range)	**9.0** (7.2–10.1)	**7.4** (5.5–8.5)	**7.5** (5.6–9.3)	**7.4** (5.2–9.8)	<0.0001	<0.0001	0.0038
Platelets 10^9^/mL; median (range)	**239** (121–345)	**358** (222–523)	**331** (170–562)	**275** (161–665)	<0.0001	<0.0001	NS

* *n for Interleukin-6 is as follows HC = 17, GCA = 40, PMR = 29, INF = 13. HC, healthy control; INF, infection control; TAB, temporal artery biopsy; FDG-PET-CT, 18F-fluorodeoxyglucose-positron emission tomography-computed tomography; IL-6, interleukin-6; Hb, hemoglobin; NS, not significant*.

### Follow-Up and Treatment

GCA and PMR patients were prospectively followed for a median period of 30 (range 0–71) and 46 months (range 0–75), respectively. For nine patients, only a pre-treatment visit could be included. We did not exclude these patients to make the pre-treatment data stronger. The number of GCA patients followed for 1 year was 31 (72%) and the number of GCA patients followed for 2 years was 23 (54%). For PMR the number of patients followed for 1 year was 24 (83%) and the number of patients followed for 2 years was 20 (69%). Patient visits were planned according to a fixed protocol. For analysis, follow-up visits were stratified into three groups: treatment phase I (2, 6 weeks, and 3 months), treatment phase II (6 and 9 months) and treatment phase III (12 months and thereafter every 6 months). All patients in treatment phase I, II and III still receive treatment. GCA patients started with a higher daily GC dose than PMR patients (median 60 mg in GCA, 15 mg in PMR). GCs were tapered upon remission according to BSR guidelines for GCA (31) and for PMR (32). In this study, a relapse is defined by GCA- or PMR-specific signs and symptoms. In case of a relapse, an extra visit to the outpatient clinic was scheduled; daily GC dose was increased and/or a conventional synthetic DMARD (methotrexate or leflunomide) was added to the treatment regimen. None of the patients in this study used IL-6 receptor blockade (e.g., tocilizumab). In patients that remained in remission, GC and/or DMARD treatment was tapered until treatment-free remission was achieved. In order to analyse stable treatment-free remission, we excluded samples from the first 3 months of treatment-free remission and hereafter only included samples of patients who did not show return of signs and symptoms for at least 6 months.

### Laboratory Measurements

Basic laboratory measurements of CRP, ESR, Hb, and platelets as well as blood leukocyte counts were collected at all available time points. CRP levels were determined using the Cobas 8000 modular analyser (Roche, Basel, Switzerland). ESR (Westergren method) and Hb were determined by the XN-9000 (Sysmex, Kobe, Japan). Platelets, monocytes and neutrophil counts were also determined by the XN-9000, based on size and granularity (diff). Levels of serum IL-6 (standard curve range 4.8–1,154; sensitivity 1.7 pg/ml) were measured with Human premix Magnetic Luminex screening assay kits (R&DSystems, Abingdon, UK) only pre-treatment [see previous study (12)]. Absolute counts of lymphocyte subsets were measured in EDTA blood by the BD (San Jose, CA, USA) MultiTest TruCount method, as described by the manufacturer. Lymphocytes were gated by size and positivity for CD45, after which the subsets were defined: CD4+ T-cells (CD3+CD4+), CD8+ T-cells (CD3+CD8+), B-cells (CD19+), and NK-cells (CD56+ and/or CD16+). TruCount measurements were performed on a FACS Canto-II (BD) and subsequently analyzed with FACSCanto Clinical Software. Monocyte counts were also determined by the TruCount method, which is based on size, granularity, and CD45 expression. We determined that counts of monocytes were 22% higher when measured by the XN-9000 method compared with the TruCount method. This factor was stable throughout all samples. Comparison of 20 samples measured by both methods and corrected (x 1.22), showed a strong correlation (*r* = 0.87, *p* = <0.0001) and good agreement on a Bland-Altman plot (Supplementary Figure 1). For this reason we applied this correction to all monocyte count measurements assessed by the TruCount method.

### Statistical Analysis

To analyse differences between groups and over time, 2-tailed non-parametric tests were performed. Fisher's exact test, Kruskal Wallis, and Mann Whitney *U*-tests were used when comparing patients with controls. Strength and statistical significance of correlations between measurements was tested using Spearman's rank correlation. The log rank test was used to compare the time to GC-free remission between patients with low or high inflammatory markers or cell counts pre-treatment. Analyses were performed with IBM SPSS 23 and GraphPad Prism 7.02 software.

## Results

### Pre-treatment: Altered Levels of IL-6, CRP, ESR, Hb, and Platelets in GCA and PMR Patients

IL-6, CRP, ESR, and platelet counts were significantly higher whereas Hb levels were significantly lower for pre-treatment GCA and PMR patients compared to HCs (Table 1). IL-6, CRP, ESR, and Hb did not differ between GCA or PMR patients and infection controls (INFs), but platelet counts were significantly higher in GCA (*p* = 0.008) and PMR (*p* = 0.033) than in INFs. Smoking status did not differ between patients groups. In GCA, CRP, and ESR correlated positively (Rho = 0.80), whereas no correlation was observed in PMR (Rho = 0.36, NS; Supplementary Figure 2). In addition, hemoglobin (Hb) correlated negatively with ESR in both patient populations (GCA Rho = −0.51, PMR Rho = −0.65). In GCA patients, platelet counts correlated positively with CRP and ESR (Rho = 0.49 and Rho = 0.54, respectively), and negatively with Hb (Rho = −0.39).

### Leukocyte Subsets in Pre-treatment GCA and PMR Patients: Shift to the Myeloid Lineage

**Figure 1:**
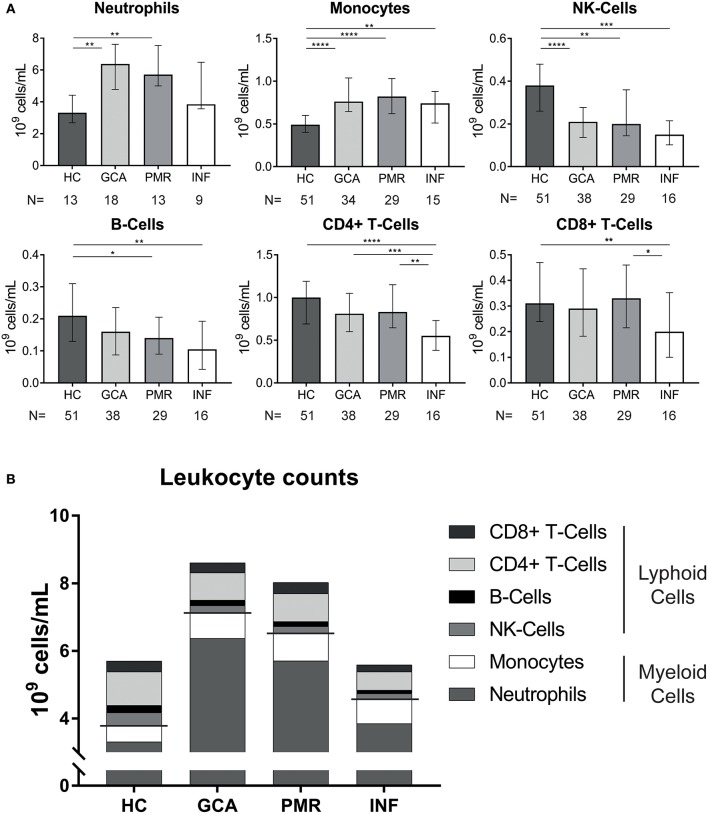
Pre-treatment measurements in newly-diagnosed, treatment naive GCA and PMR patients. **(A)**, Leukocyte counts in the blood for GCA and PMR as well as two control groups: HC and INF. The n is depicted in the figure and indicates the number of samples measured in the different groups. Data is expressed as median and interquartile range. Statistical differences by Mann Whitney *U* between groups are displayed if Kruskal Wallis testing indicated significant differences: *p < 0.05, **p < 0.01, ***p < 0.001,****p < 0.0001. **(B)**, Stacked leukocyte subset counts show a clear shift to the myeloid lineage in GCA and PMR pre-treatment.

**Figure 2:**
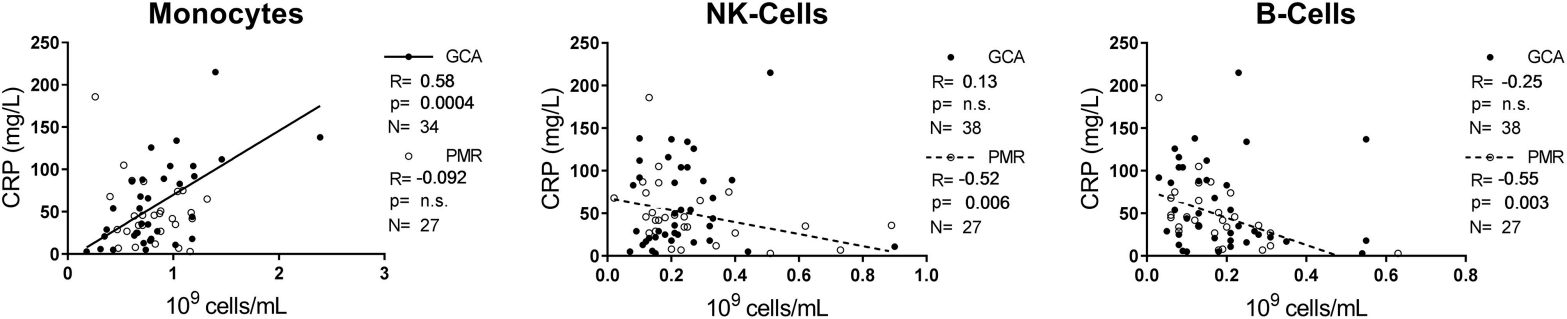
Correlations between three leukocyte subsets and the inflammatory marker CRP. Correlations between CRP and the leukocyte subset in pre-treatment GCA (closed circles) and PMR (open circles) patients. Spearman's *R*, the *p*-value of the correlation and the N are indicated in each graph for GCA and PMR. Regression line for GCA is shown as an uninterrupted line, for PMR as a dotted line. Correlations for neutrophils, CD4+ T-cells, and CD8+ T-cells are displayed in Supplementary Figure 2.

Absolute counts of leukocyte subsets measured in GCA and PMR patients before start of treatment were compared with counts in HCs and INFs (Figure 1A). Counts of neutrophils and monocytes were significantly higher, while NK-cells were significantly lower in GCA and PMR patients compared to HCs. Counts of these subsets in patients were similar to those in INF. B-cell counts were also significantly lower in PMR while for GCA patients a trend toward reduction of B-cells was observed (*p* = 0.06). In contrast, T-cells (both CD4+ and CD8+) in GCA and PMR patients were not significantly different from HC, even though T-cell counts were lower in INFs. Overall, we observed a shift in leukocyte counts toward the myeloid lineage in both GCA and PMR patients as myeloid cell counts were elevated, while lymphoid cell counts were reduced or unchanged (Figure 1B). To determine a possible involvement of leukocyte subsets in disease activity, we correlated numbers of circulating leukocyte subsets with CRP levels determined at the same visit (Figure 2). A significant positive correlation was observed between monocyte counts and CRP in GCA-patients only (Rho = 0.58), whereas CRP from PMR patients correlated negatively with numbers of circulating B-cells (Rho = −0.55) and NK-cells (Rho = −0.52). In addition, CRP from INFs showed a strong correlation with neutrophil counts (Rho = 0.70). For all correlations, see Supplementary Figure 2.

### During Treatment: Myeloid Subsets Remain Elevated Whereas Lymphoid Subsets Fluctuate

**Figure 3:**
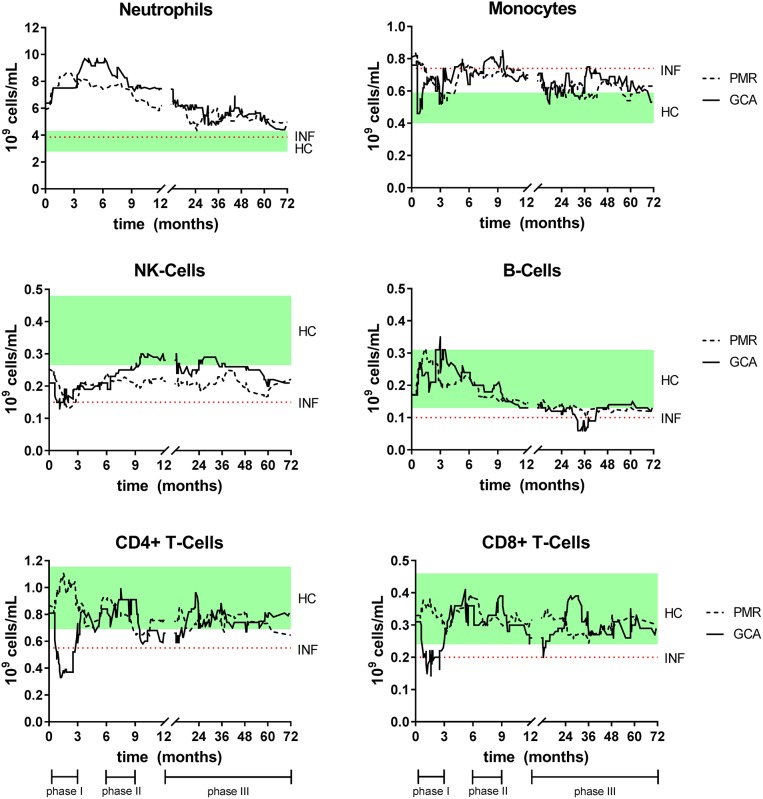
Smoothed median of leukocyte counts for GCA and PMR patients over time while on GC treatment. The smoothed median is calculated by taking the median of each new measurement and that of the 19 measurements before that point. This method enables to distinguish patterns over time that would be unnoticeable if each point is plotted separately. For interpretation the interquartile range of HC (green box, cross-sectional measurement) and the median of the INF (dotted line, cross-sectional) were added to the figures. Time point 0 indicates the pre-treatment sample. Also, the three different treatment phases are indicated.

**Figure 4:**
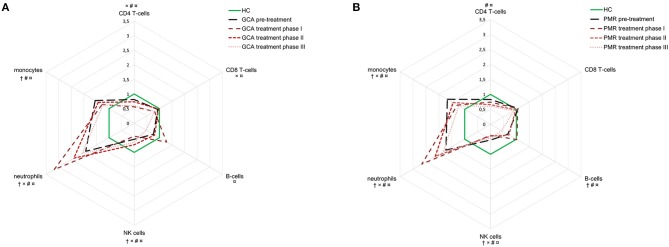
Dynamics in cell population counts during follow-up in GCA **(A)** and PMR **(B)** patients. Counts are expressed in radar plots as median fold-change compared to healthy controls (*n* = 51) for the following groups: pre-treatment (GCA *n* = 42, PMR *n* = 31), treatment phase I (GCA *n* = 38, 69 measurements; PMR *n* = 25, 54 measurements), phase II (GCA *n* = 32, 43 measurements; PMR *n* = 23, 33 measurements), and phase III (GCA *n* = 29, 65 measurements; PMR *n* = 19, 56 measurements). Pre-treatment only includes the visit before start of treatment; treatment phase I includes follow-up visits at 2, 6 weeks, and 3 months; treatment phase II includes 6 and 9 months; treatment phase III includes 12 months and beyond. †: sign difference between HC and baseline, ×: sign difference between HC and treatment phase I, #: significant differences between HC and phase II, and ¤: significant differences between HC and phase III (Mann-Whitney *U*-test, *P* < 0.05).

After diagnosis and pre-treatment sampling, all patients started with GC treatment. To visualize fluctuations in absolute leukocyte counts during follow-up in both GCA and PMR, a (smoothed) median of 20 consecutive measurements over time was calculated and depicted in Figure 3. To apply an appropriate statistical analysis of treatment effects over time and to compare it with the HC group, the follow-up time was split into three treatment phases as depicted in Figures 3, 4. Median daily GC dose successively decreased for GCA patients in treatment phase I, II, and III: 40, 10, and 5 mg, respectively. In PMR patients this was 15, 7.5, and 5 mg. In both GCA and PMR patients on treatment, myeloid cell counts (monocytes and neutrophils) remained higher over time compared to HCs (Figures 3, 4). Neutrophils increased further during treatment phase I and II when compared to pre-treatment. Lymphoid cells (NK-, T-, and B-cells) were also affected by treatment. B-cells showed most fluctuations over time: during treatment phase I, we observed an increase compared to pre-treatment which was followed by a progressive decrease in treatment phase II and III. T-cell (CD4+ and CD8+) counts were low during treatment compared to pre-treatment and to HC counts. Interestingly, T-cell counts dropped significantly during treatment phase I in GCA patients, while this was not observed in PMR patients where T-cells were only lowered in phase II and III. NK-cells remained significantly lower throughout all phases compared to HCs. Platelets, CRP, and ESR all decreased from pre-treatment levels during the entire treatment but mostly remained elevated when compared to HCs (Supplementary Figure 3). In GCA patients platelets were elevated in all phases, but CRP and ESR were elevated only in phase II and III. In PMR patients platelets were elevated in phase I, CRP in phase I and II and ESR in phase II and III. Hb increased from pre-treatment levels in both GCA and PMR but remained decreased compared to HC levels during all phases.

### ESR Outperforms CRP, Hb, and Platelets as Marker for Relapses in GCA During Treatment

**Figure 5:**
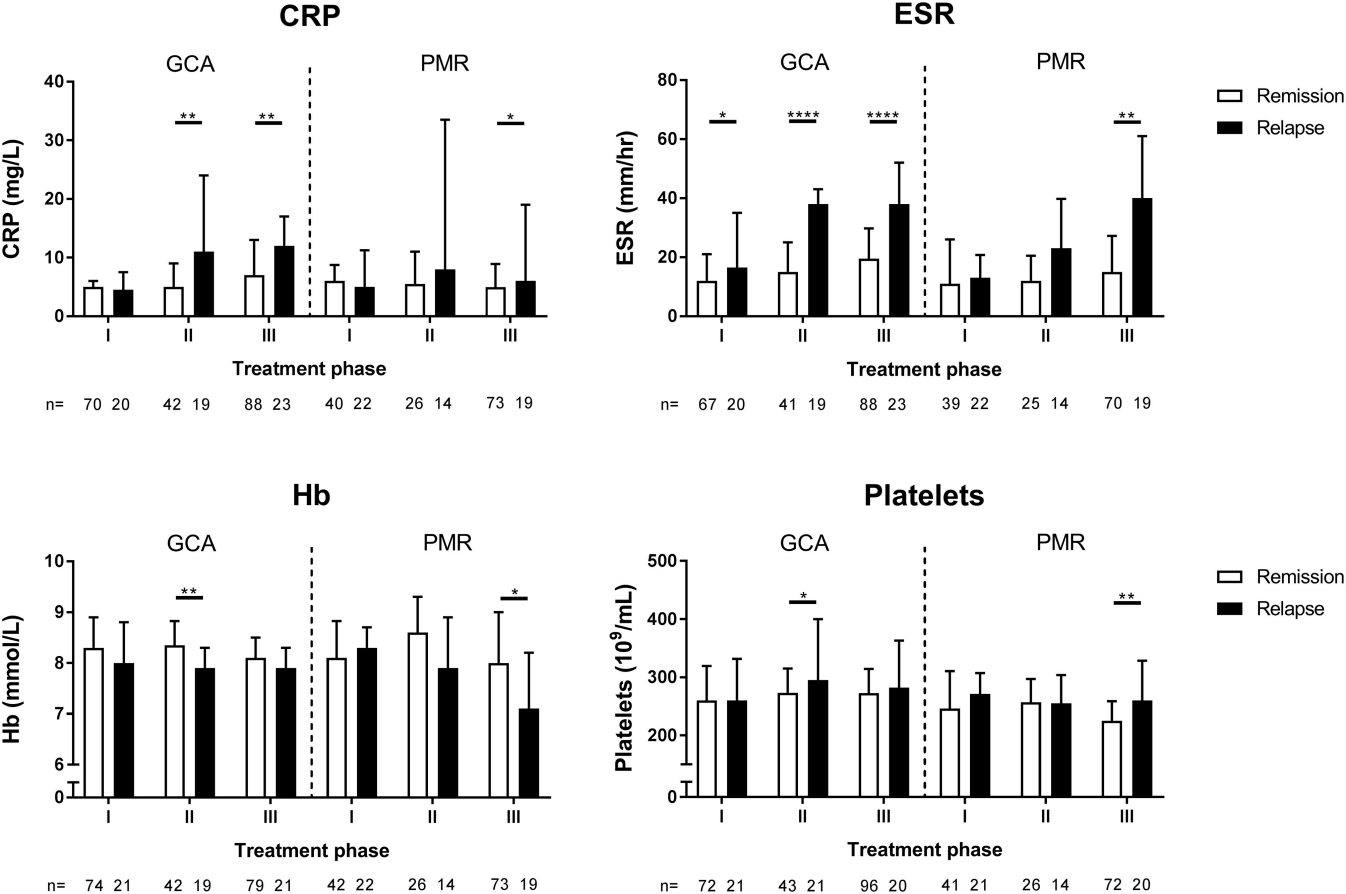
Levels of inflammatory markers during treatment phase I, II, and III for GCA and PMR patients in remission and during relapse. The definition of remission and relapse was based solely on clinical signs and symptoms. Data is expressed as median plus interquartile range. The number of measurements is indicated by *n*. Statistical significance is expressed as follows: *p < 0.05, **p < 0.01, ****p < 0.0001 (Mann Whitney *U*-test).

In order to determine the GC dose at which patients experienced a relapse, the daily GC dose of patients who were followed for at least 2 years was recorded (Table 2). Of the 24 GCA patients fulfilling this criterion, 79% experienced at least one relapse during these 2 years. In addition, 57% of the 21 PMR patients developed at least one relapse during this period. The median daily dose at relapse was 5 mg for GCA and 7.5 mg for PMR. Six GCA relapses and five PMR relapses occurred in patients who were not taking GCs anymore. Next, we determined whether levels of inflammatory markers and leukocyte counts were different in relapsing patients compared to patients in treatment-induced remission (Figure 5). CRP levels did not reflect relapses in treatment phase I for both GCA and PMR patients when compared to remission patients. In PMR this was also true for treatment phase II. ESR, however, did discriminate GCA patients experiencing a relapse from remission patients in all treatment phases. In PMR patients this was only the case for treatment phase III. Lower Hb and higher platelets were observed in relapsing GCA patients during phase II and in relapsing PMR patients during phase III. There were also differences in leukocyte counts between relapsing and remission patients in PMR. During relapses, patients displayed higher CD4+ T-cells in treatment phase I, lower NK-cells in phase II, and higher neutrophils in phase II and III (data not shown).

**Table 2:** Daily glucocorticoid dose use at the time of relapse for GCA (*N* = 24) and PMR (*N* = 21) patients.

**GC dose (mg/day) at relapse**	**GCA relapses (%)**	**PMR relapses (%)**
0	6 (21)	5 (25)
1–5	10 (35)	3 (15)
6–10	7 (24)	6 (30)
11–20	6 (21)	5 (25)
>20	0	1 (5)
Total	29	20

*In patients that were followed for 2 years, we registered the daily GC dose at which they relapsed. Relapses were defined by clinical signs and symptoms only. GC, glucocorticoid; GCA, giant cell arteritis; PMR, polymyalgia rheumatica*.

### The Myeloid and Inflammatory Profiles Persist in Treatment-Free Remission Patients

**Figure 6:**
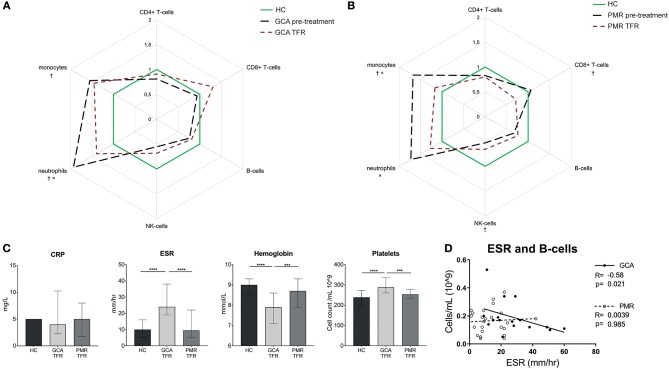
Different leukocyte subset counts and inflammatory markers in treatment- free remission. Leukocyte subset counts pre-treatment and in treatment-free remission **(A:** GCA *n* = 13 patients, 17 samples and **B:** PMR *n* = 15 patients, 25 samples) were expressed as median fold-change compared to healthy controls. †: sign difference between HC and treatment-free remission. ×: sign difference between pre-treatment and treatment-free remission (Mann-Whitney *U*-test *p* < 0.05). **(C)** Inflammatory markers in HC, GCA treatment-free remission, and PMR treatment-free remission (Mann-Whitney *U*-test: ***p < 0.001,****p < 0.0001). Data is expressed as median and interquartile range. **(D)** Correlation between B-cell counts and ESR in treatment-free remission patients.

To determine whether leukocyte counts and inflammatory markers of GCA and PMR patients are truly normalized after treatment cessation, we investigated patients in treatment-free remission (defined as 3 months treatment-free and in stable remission for the next 6 months). So far, 13 GCA and 15 PMR patients have reached treatment-free remission and were included in the analysis. GCA patients in treatment-free remission showed persistently elevated myeloid cell counts compared to HCs. Note that, compared to pre-treatment levels, neutrophil counts were found reduced (Figure 6A). PMR patients in treatment-free remission also still demonstrated significantly elevated myeloid cell counts although monocyte counts had decreased since pre-treatment levels (Figure 6B). Furthermore, NK-cell and CD8+ T-cell counts were lower in PMR treatment-free remission patients than in HCs. In GCA, there was a strong trend toward lower NK-cells in treatment-free remission compared to HCs (*p* = 0.05). Inflammatory markers normalized to HC levels in PMR patients in treatment-free remission (Figure 6C). In contrast, in treatment-free remission GCA elevated ESR and platelet counts and lowered Hb remained, whereas CRP was normal. We further investigated whether the elevated ESR in GCA patients was linked to changes in leukocyte subsets. We found a strong negative correlation between B-cell counts and ESR in treatment-free remission GCA patients (Figure 6D).

### Pre-treatment Low Hb Predicts Longer GC Requirement in PMR

**Figure 7:**
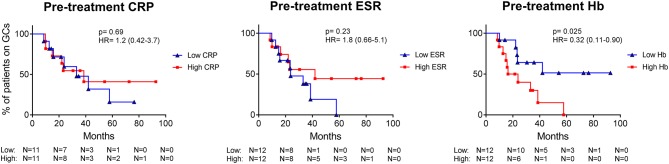
In PMR patients, long-term GC requirement is predicted by pre-treatment Hb levels, but not by CRP and ESR. The CRP, ESR, and Hb of PMR patients before treatment were split into low or high levels (based on the median) and were plotted in a Kaplan-Meier curve against time to GC-free remission. *p*-value and hazard ratio (HR; including 95% confidence interval) of the log rank test are depicted in the graphs.

Finally, we assessed whether leukocyte subset counts and inflammatory markers, assessed before start of treatment, could predict time to GC-free remission. A predictive factor was found in PMR patients, only. Pre-treatment Hb level higher than the median, predicted a short time to GC-free remission (i.e., a favorable disease course) compared to patients with a low Hb before start of treatment (Figure 7, *p* = 0.025). As the Hb is typically higher in males, we checked the sex distribution between PMR patients with low and high Hb and found an exact equal distribution. The other inflammatory markers, CRP, and ESR, were not prognostic for GC requirement, nor were any of the leukocyte subset counts.

## Discussion

This prospective study provides a comprehensive overview of peripheral blood leukocyte dynamics and inflammatory markers in GCA and PMR during the entire disease course: before and after start of glucocorticoid treatment as well as in stable treatment-free remission. Our main finding is that leukocyte counts shift to the myeloid lineage in both GCA and PMR and that this myeloid bias persists in spite of GC treatment and extends well into treatment-free remission.

Counts of myeloid leukocyte subsets were elevated in pre-treatment GCA and PMR patients. This may be explained by the actions of IL-6, a key pro-inflammatory cytokine in GCA and PMR, promoting monocyte and neutrophil production in the bone marrow (33). In contrast to myeloid subset counts, pre-treatment lymphoid subset counts were either lowered (NK- and B-cells) or unchanged (CD4 and CD8 T-cells). In INF all lymphoid cell counts were lowered. These findings are in accordance with the notion that inflammation shifts the development of hematopoietic stem cells toward the myeloid lineage (34). Previous studies have mostly documented similar findings (10, 24–29, 35), albeit that some reported on lowered monocyte counts (26) and lowered CD8+ T-cells (26, 27). Typical acute phase markers are elevated in both GCA and PMR patients, at pre-treatment analysis, even though the ESR is significantly lower in PMR than in GCA patients. In contrast to GCA, CRP, and ESR are not correlated with each other in PMR patients. CRP is considered a more acute marker of inflammation, while ESR is more associated with longer-term chronic inflammation (36). The ESR is a composition of several proteins, including fibrinogen, Hb, and immunoglobulin levels (37). Whether there is a discrepancy in the ESR of GCA patients compared to PMR patients, remains to be investigated. Remarkably, platelet counts were found even higher than INF. Whether platelet counts are useful as disease-specific biomarker, needs to be evaluated in a larger cohort. In GCA, systemic symptoms (e.g., fever, weight loss) are linked to the IL-6-dependent acute-phase response (38). Previously, we indeed observed a strong positive correlation between IL-6 and CRP in our cohort (12). In the current study, we also found pre-treatment CRP to be positively correlated with monocyte counts in GCA patients. Monocytes are important in the immunopathogenesis of GCA and work in tandem with CD4+ T-cells to promote granulomatous inflammation, angiogenesis, and destruction of the vessel wall (5, 39). Monocytes, as part of the innate immune system, sense pathogens, and danger signals by pattern recognition receptors, including toll-like receptors (TLRs) (40). Previously, TLR7 expression on monocytes of GCA and PMR patients was found elevated, hinting at a higher responsiveness to viral antigens (41). The chemokine CCL2 is important for monocyte migration, and its levels were found lower in the blood of GCA patients (25). This could be explained by the usage of CCL2 by monocytes migrating from the bone marrow to the blood. In pre-treatment PMR, we found a negative correlation of CRP with B-cells and NK-cells, hinting that these cell types are important in the maintenance of immune homeostasis. This could be through immune regulatory functions as described before for both subsets (38, 39, 42, 43). Alternatively, low B-cell and NK-cell counts may reflect tissue migration. It is currently unknown if B-cells or NK-cells infiltrate PMR synovia but B-cell counts were found to be decreased in GCA and B-cells and are present in GCA vessels (8, 9, 44), implying migration. In GCA, NK-cells are not frequently found in the TABs, arguing against migration (25).

In this study, we also chartered effects of treatment on leukocyte subsets and inflammatory markers in GCA and PMR patients over time. Blood counts of monocytes and neutrophils remained elevated in patients compared to controls throughout the entire treatment period. GC-induced leukocytosis is a well-known phenomenon (45) and is mainly due to the effect of GCs on neutrophils. GC treatment causes the release of neutrophils from the marginal pool by decreasing the expression of adhesion molecules Mac-1 and L-selectin needed to bind to the endothelium (46, 47). GCs mainly increase counts of mature neutrophils in the blood, as influx of infection-related, “non-segmented” neutrophils from the bone marrow is minimal (45, 47). While monocyte counts remained elevated compared to HC levels, they were lowered by GC treatment. This is likely due to a decrease in non-classical monocytes which are sensitive to GC-induced apoptosis (25, 48). GC-treatment affected lymphoid leukocyte counts as well. Interestingly, a difference between GCA and PMR in CD4+ T-cell counts was observed in the first months of treatment, as these counts were markedly decreased in GCA patients only. This is likely caused by GCA patients receiving a higher GC dose. Indeed, high- but not low-dose GCs, have a strong apoptotic effect on CD4+ T-cells *in-vitro* and *in-vivo* and this decrease is associated with inhibition of IL-2 signaling (49). Also noticeable is the pattern of B-cells during treatment in GCA patients; early treatment led to an increase in B-cell counts. This is likely caused by B-cells returning to the circulation from peripheral sites (9). In our cohort, patients on long-term GC treatment became lymphopenic, as especially their CD4+ T-cell and B-cell counts gradually lowered over time. In addition, NK-cell counts, that were already lower pre-treatment, were not found to reduce further on treatment. This is in accordance with previous reports on NK-cell counts in GCA and PMR and in line with NK-cells being resistant to GC-induced apoptosis (26, 29, 49). Overall, long-term GC use significantly changes the composition of the peripheral leukocyte pool as well as the function of leukocyte subsets (14, 15), thereby making GCA and PMR patients susceptible to infections (50).

Our data are in congruence with the notion that GCs manage to actively suppress symptoms of the disease but have only a partial effect on tissue inflammation. This is based on the inflammation-induced myeloid dominance observed before treatment that persisted during treatment, despite a suppressed CRP and ESR. Indeed, a too rapid tapering of GCs will in most cases lead to a return of signs and symptoms (50, 51). The observed GC dose at which patients experience their first relapse is in line with previous reports (52). Moreover, a study investigating sequential TABs revealed that at least 44% of GCA patients have persistent inflammation in spite of treatment-induced remission (22). Furthermore, recent studies on tissue inflammation markers during tocilizumab treatment raise caution for ongoing inflammation despite absence of symptoms (21, 53).

The strong suppressive effect of GCs on the acute-phase response makes the classic inflammatory markers, CRP and ESR, less trustworthy for monitoring disease activity. In the first months of treatment, solely ESR discriminated between relapsing and remission GCA patients and the difference in ESR between these groups were found to become significantly stronger at later phases and thus at lower GC doses. Overall, ESR appeared more suited than CRP in identifying relapses in GCA rendering ESR more useful in monitoring disease activity. This is in accordance with a previous study on monitoring biomarkers in GCA (54). However, as described before (55), CRP and ESR are frequently normal at time of clinical relapse. This unsatisfactory use of CRP/ESR during GC treatment, and the fact that tocilizumab treatment suppresses these markers even more (21), raises the need for new inflammatory markers to aid in monitoring of GCA and PMR patients. Peripheral blood cells of GCA and PMR patients in treatment-free remission were found to retain the myeloid bias. This may be explained by a long-lasting imprint of inflammation on peripheral blood cell composition. Yet, whereas markers of inflammation normalized in PMR, these markers (ESR, Hb, and platelet counts, but not CRP) remained altered in GCA patients that have reached treatment-free remission. The combined data suggest that subclinical vessel wall inflammation may still be ongoing in GCA. Alternatively, this retained myeloid dominance could point toward cellular senescence of the immune system which had predisposed the patients to develop these diseases. Indeed, aging of the immune system has been linked to development of disease (56). Interestingly, this ongoing response (ESR) is negatively correlated with B-cell counts. B-cells might be important in preventing a return of disease and/or B-cells might aggravate disease by tracking to the site of inflammation. Migration of B-cells toward the inflamed vessel has been documented in GCA (7, 8, 44) but their role in the tissue (either anti-or pro-inflammatory) remains to be established. Thus, the question remains whether symptom treatment of GCA (and PMR) is sufficient. Persistence of the myeloid and inflammatory profile suggests ongoing inflammation eventually leading to vascular damage and associated morbidity and mortality (57). Additionally, we discovered a prognostic value of pre-treatment Hb levels on disease course in PMR patients. Our data show that patients with a low Hb have a higher risk for an unfavorable long-term disease course. No such prognostic value was seen for ESR and CRP. The low pre-treatment level of Hb in PMR patients is a secondary effect of long-term inflammation (58). We thus hypothesize that a low Hb better reflects the inflammatory load over a longer period of time than ESR and especially CRP. The latter inflammatory markers are indeed more prone to fluctuate over time (36). The clinical utility of our finding is that low Hb levels may predict long-term GC requirement in PMR patients. The major strength of this study is our well-defined, prospectively followed, long-term cohort of GCA and PMR patients who joined the study when they were treatment-naive, allowing to assess pre-treatment values. Often, GCA and PMR patients are included in cohorts after start of GCs. The strict follow-up regimen allowed us to investigate the immune status of patients during relapses and in treatment-free remission. Because of the clinical overlap between the two diseases, the drawn comparisons in this study are useful. The inclusion of the INF group helped to discriminate between disease specific and non-specific features. Another strength of the study is that we documented changes in six major peripheral blood leukocyte subsets using assays that are readily available in the clinical setting. Our study is limited by sole analysis of peripheral blood markers in both these systemic diseases which may only partly mirror the immunological processes at the sites of inflammation such as the vessel wall and the synovium in GCA and PMR, respectively. In conclusion, we observed a clear shift toward the myeloid lineage in pre-treatment GCA and PMR patients. This myeloid bias was associated with inflammatory markers and persisted during glucocorticoid treatment and in treatment-free remission. Persistence of the myeloid and inflammatory profile during the entire disease course may reflect ongoing subclinical vasculitis, implying that current glucocorticoid-based treatment is unsatisfactory. Future studies using sensitive imaging techniques should address if these profiles indeed coincide with tissue inflammation. Also, treatment could aim at targeting the myeloid shift in GCA and PMR patients. Blocking the granulocyte macrophage colony stimulating factor (GM-CSF) receptor could prove to be beneficial in influencing this shift. Trials with this type of treatment are currently ongoing (NCT03827018).

## Data Availability

The raw data supporting the conclusions of this manuscript will be made available by the authors, without undue reservation, to any qualified researcher.

## Ethics Statement

This study was carried out in accordance with the recommendations of the institutional review board of the University Medical Center Groningen with written informed consent from all subjects. All subjects gave written informed consent in accordance with the Declaration of Helsinki. The protocol was approved by the institutional review board of the University Medical Center Groningen.

## Author Contributions

YvS, JG, AB, and EB conceived and designed the study. YvS and JG acquired data. All authors were involved in data analysis and/or interpretation. YvS, JG, and MS drafted the manuscript and all authors revised it critically for important intellectual content. All authors gave final approval of the version to be published and agree to be accountable for all aspects of the work in ensuring that questions related to the accuracy or integrity of any part of the work are appropriately investigated and resolved.

### Conflict of Interest Statement

AB was a consultant for Gruenenthal Gmbh until 2017. WA and EB have received funding from the European Union's Horizon 2020 research and innovation program (RELENT). EB as an employee of the UMCG received speaker fees and consulting fees from Roche which were paid to the UMCG. KvdG work was supported by the Dutch Society for Rheumatology (Rheumatology grant 2017) and the UMCG Mandema Stipend. The remaining authors declare that the research was conducted in the absence of any commercial or financial relationships that could be construed as a potential conflict of interest.
