# Supplementary material for: Leukocyte Dynamics Reveal a Persistent Myeloid Dominance in Giant Cell Arteritis and Polymyalgia Rheumatica
Source: Front Immunol. 2019 Aug 22;10:1981. doi: 10.3389/fimmu.2019.01981 (PMC6714037; doi:10.3389/fimmu.2019.01981)
Supplement: Supplementary file 1 [file Data_Sheet_1.docx]

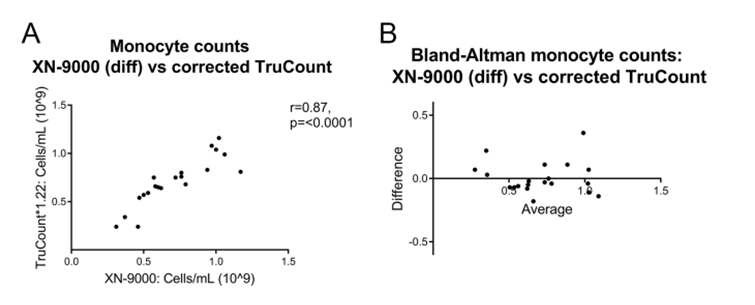


Supplementary figure 1: Comparison between two methods of measuring monocyte counts. The first method is the XN-9000 (diff) and the second method is the corrected Trucount values presented were calculated by multiplying with 1.22. **A**, Correlation between the two measurement methods for 20 samples. **B**, Bland-Altman plot showing the agreement between the two different methods for 20 samples.

**B**


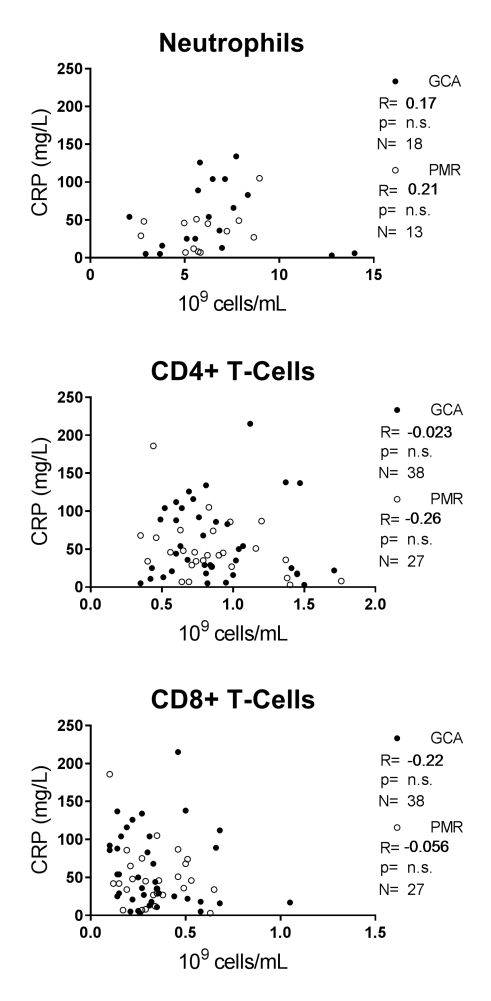

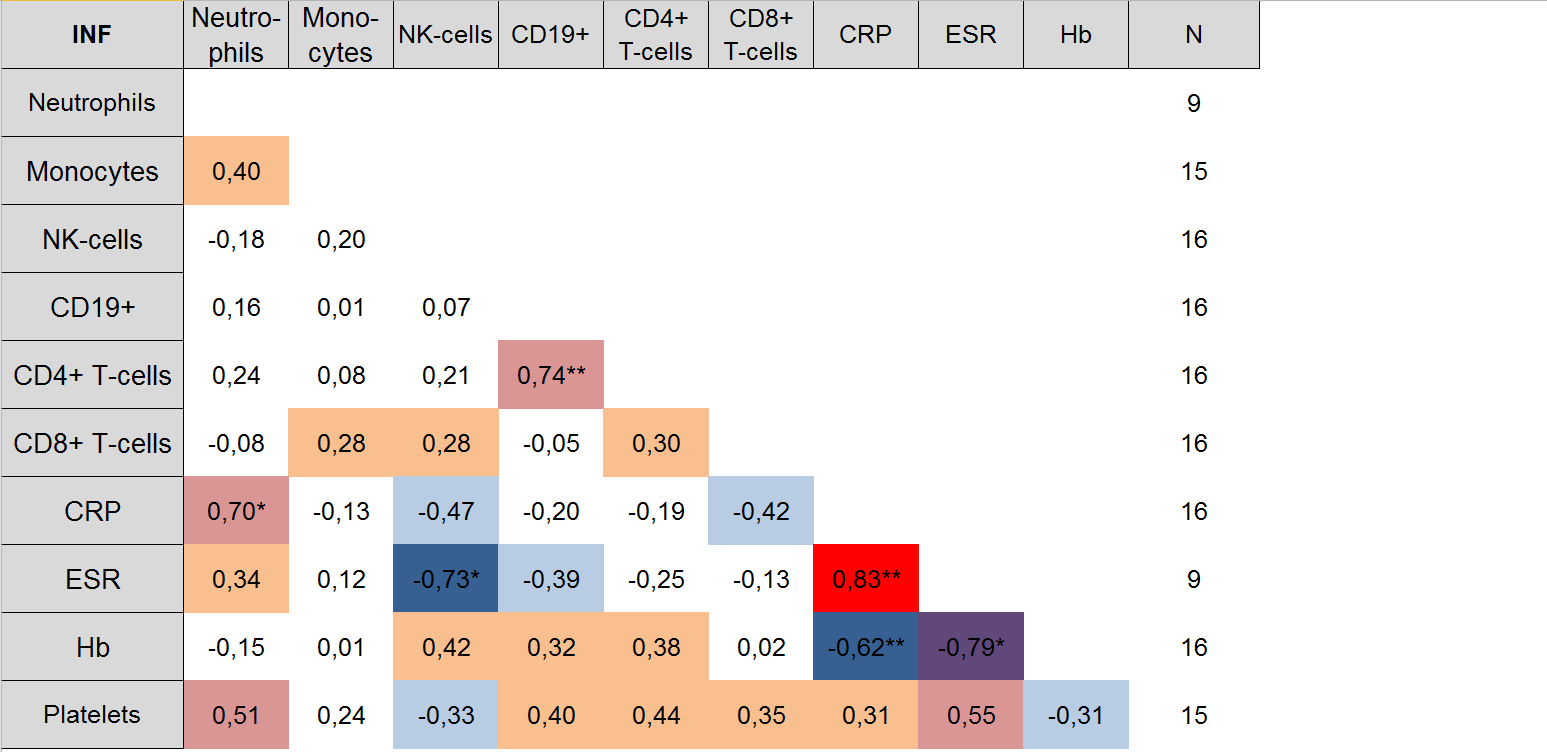

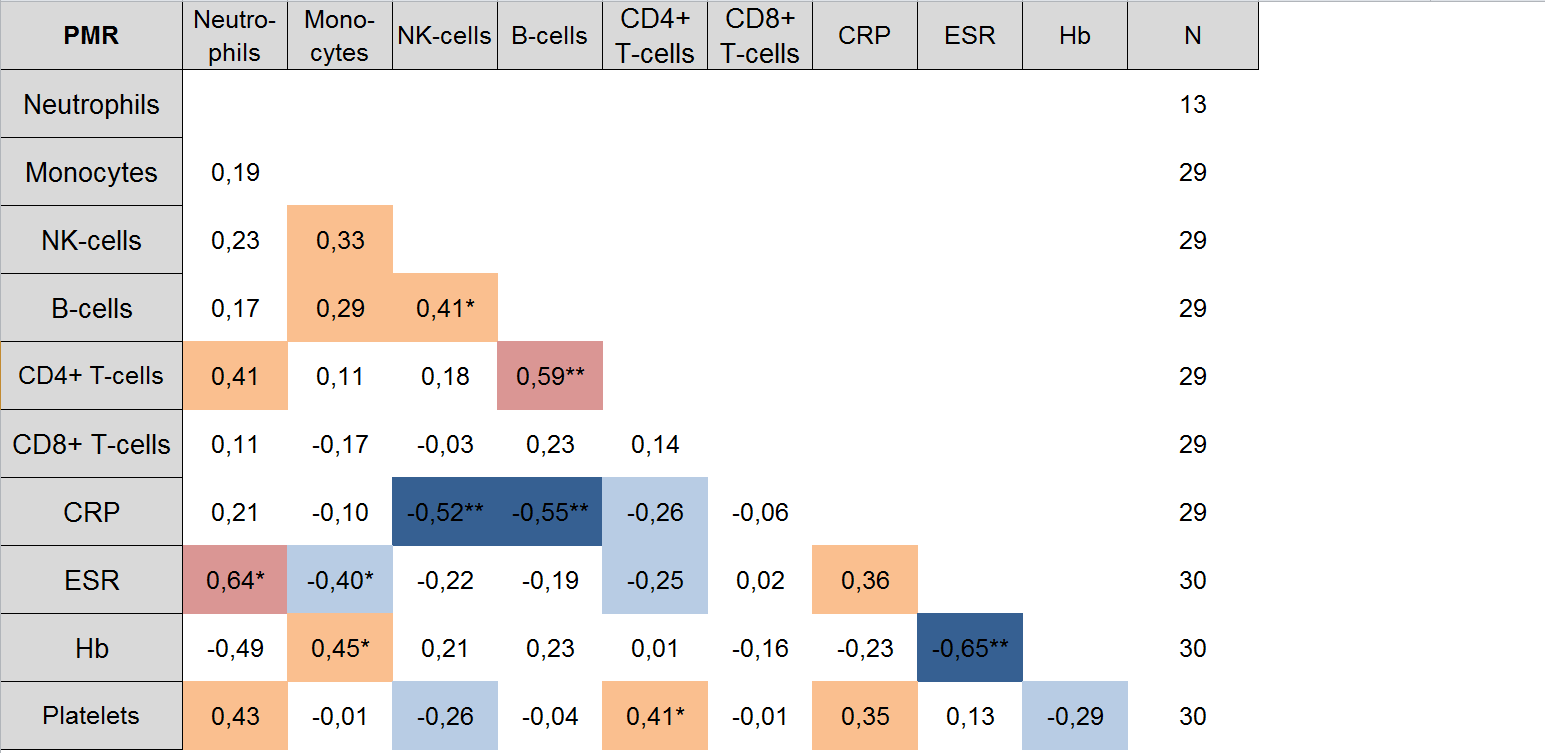

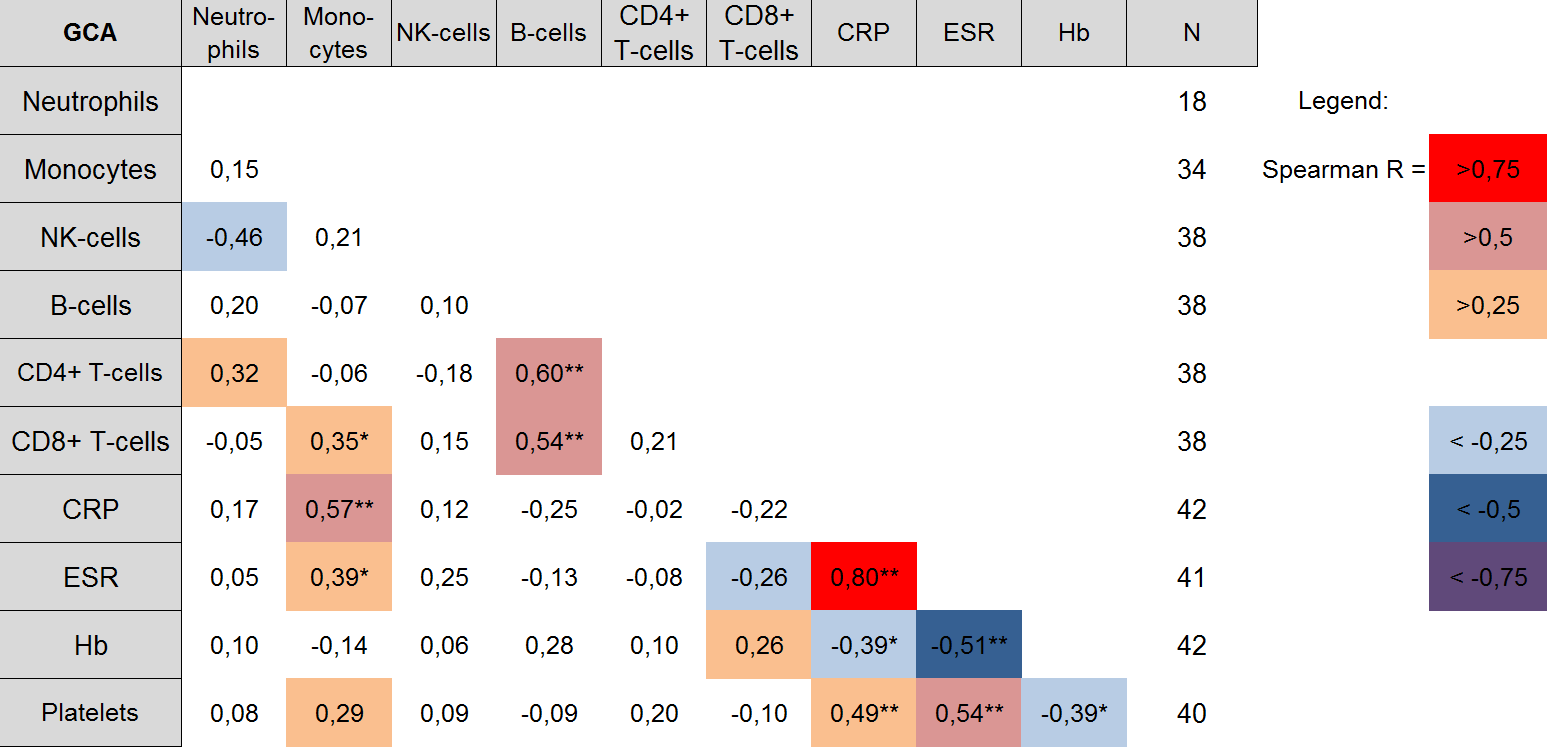


**A**

**B**

Supplementary figure 2: **A:** Spearman’s correlation coefficients for leukocyte subset counts and disease activity parameters in pre-treatment GCA (N=42) and PMR (N=31) patients, and in INF (N=16). The N in the figure depicts the number of measurements. Strength of the correlation is indicated by the cell colors. Significant correlations are flagged by * (p<0.05) and ** (p<0.01). **B:** Correlations between three leukocyte subsets and the inflammatory marker CRP. Correlations between CRP and the leukocyte subset in pre-treatment GCA (closed circles) and PMR (open circles) patients. Spearman’s R, the p-value of the correlation and the N are indicated in each graph for GCA and PMR. Regression line for GCA is shown as an uninterrupted line, for PMR as a dotted line.


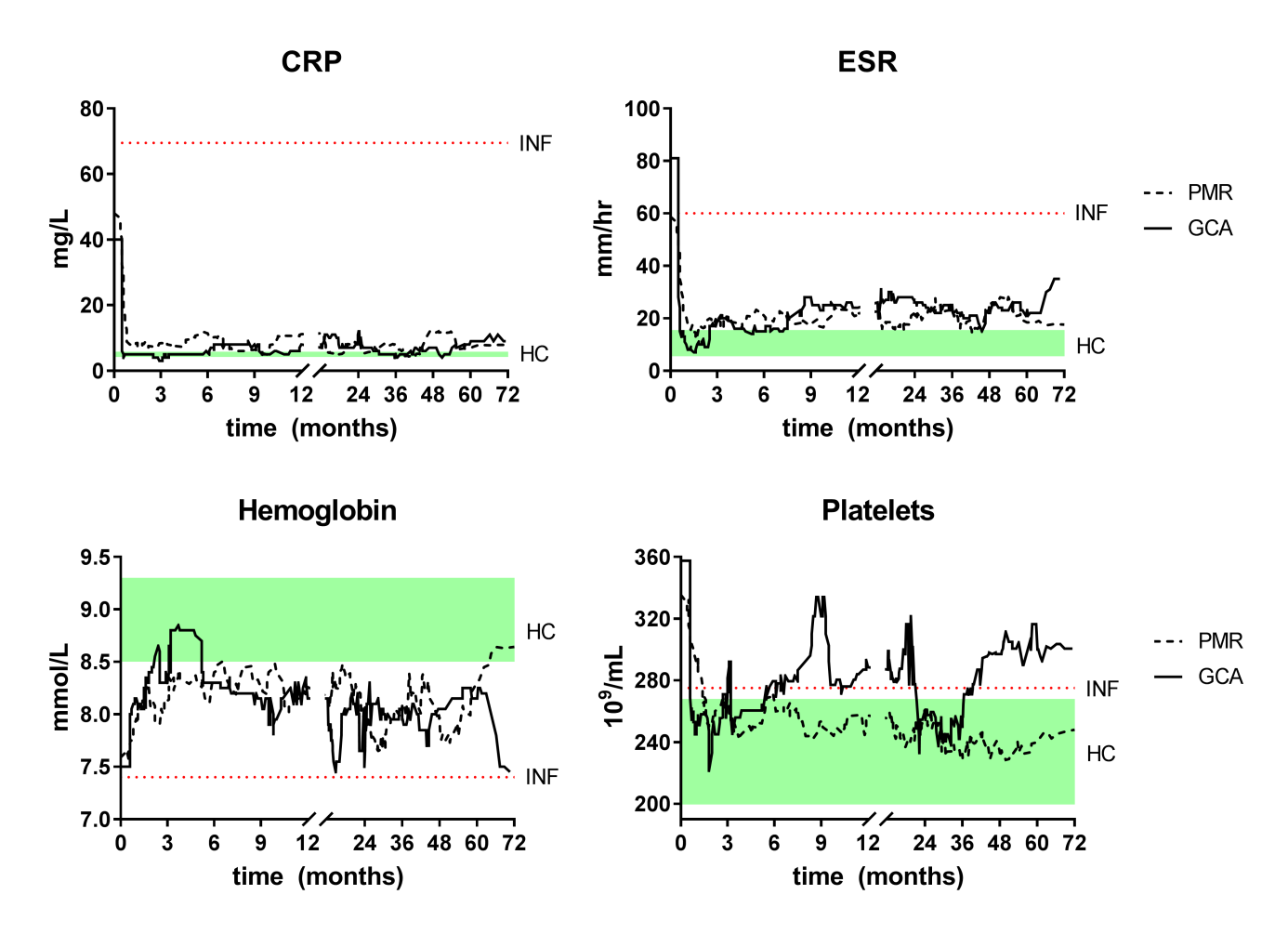
Supplemental figure 3: Moving median of disease activity markers for GCA and PMR patients over time while on GC treatment. For interpretation of the data the interquartile range of HC (green box, cross sectional measurement) and the median of the INF (dotted red line, cross sectional measurement) were added to the figures. Time point 0 indicates the pre-treatment sample. GCA: giant cell arteritis, PMR: polymyalgia rheumatica, HC: healthy control, INF: infection control.
